# Supplementary material for: Gut Microbiota-Mediated Pharmacokinetic Drug–Drug Interactions between Mycophenolic Acid and Trimethoprim-Sulfamethoxazole in Humans
Source: Pharmaceutics. 2023 Jun 14;15(6):1734. doi: 10.3390/pharmaceutics15061734 (PMC10304707; doi:10.3390/pharmaceutics15061734)
Supplement: Supplementary file 1 [file pharmaceutics-15-01734-s001.zip › Figure S1.pdf]

Supplementary Figure S1

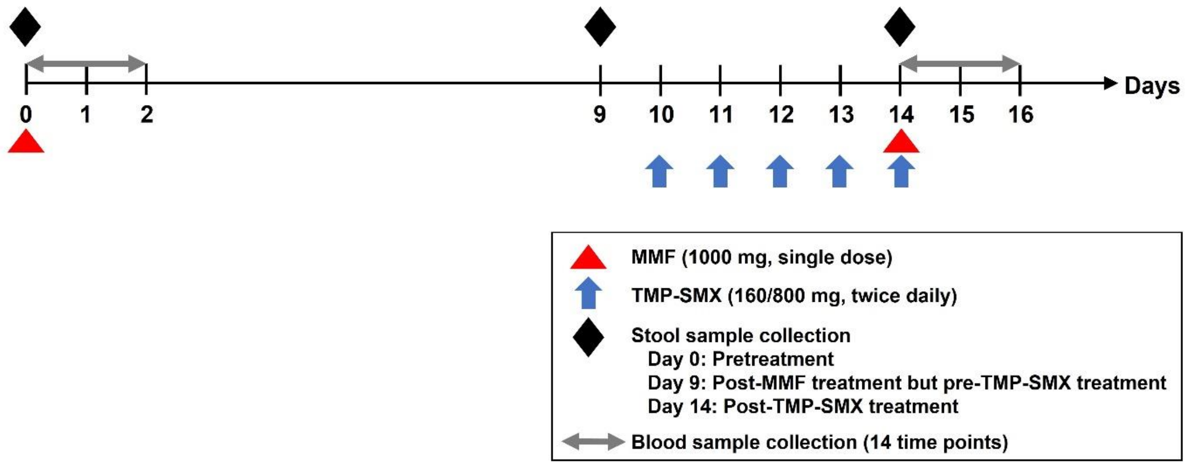

Figure S1. Study schema. Days of administration of MMF, TMP-SMX, as well as blood and stool sample collections from the study participants, are presented.
