# Supplementary material for: Gut Microbiota-Mediated Pharmacokinetic Drug–Drug Interactions between Mycophenolic Acid and Trimethoprim-Sulfamethoxazole in Humans
Source: Pharmaceutics. 2023 Jun 14;15(6):1734. doi: 10.3390/pharmaceutics15061734 (PMC10304707; doi:10.3390/pharmaceutics15061734)
Supplement: Supplementary file 1 [file pharmaceutics-15-01734-s001.zip › Figure S2.pdf]

Supplementary Figure S2

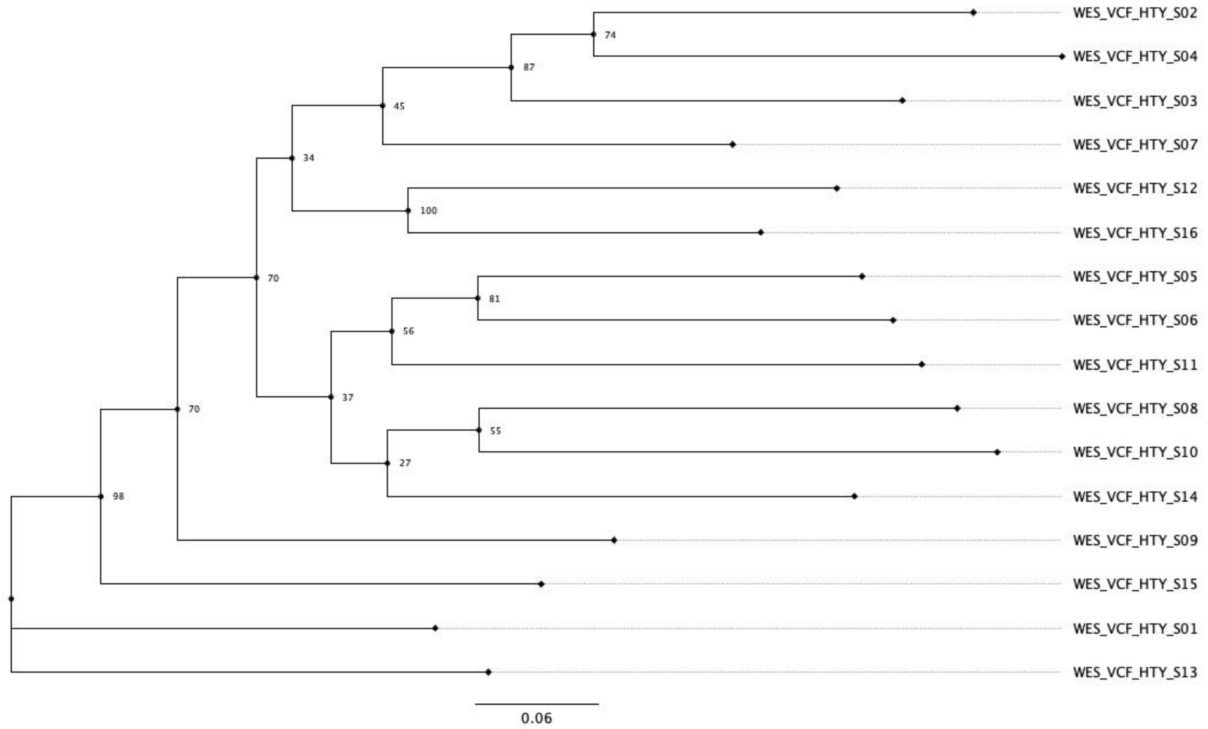

Figure S2. A phylogenetic tree constructed from SNPs detected in the 681 genes known to play roles in human gut microbiota. This unrooted tree was constructed using GTR+F+R2 as a model of nucleotide substitution. Numbers at the nodes are ultrafast bootstrap support (%). The Log-likelihood of this tree is -146167.2681 (s.e. 779.7589). Unconstrained log-likelihood (without tree) is -236719.7879. Akaike information criterion (AIC) score is 292412.5361, and a corrected Akaike information criterion (AICc) score is 292412.6342. Bayesian information criterion (BIC) score is 292738.9019. The total tree length (sum of branch lengths) is 3.9308, and the sum of internal branch lengths is equal to 0.5239 (13.3278% of tree length).
