# Supplementary material for: Gut Microbiota-Mediated Pharmacokinetic Drug–Drug Interactions between Mycophenolic Acid and Trimethoprim-Sulfamethoxazole in Humans
Source: Pharmaceutics. 2023 Jun 14;15(6):1734. doi: 10.3390/pharmaceutics15061734 (PMC10304707; doi:10.3390/pharmaceutics15061734)
Supplement: Supplementary file 1 [file pharmaceutics-15-01734-s001.zip › Figure S4.pdf]

Supplementary Figure S4

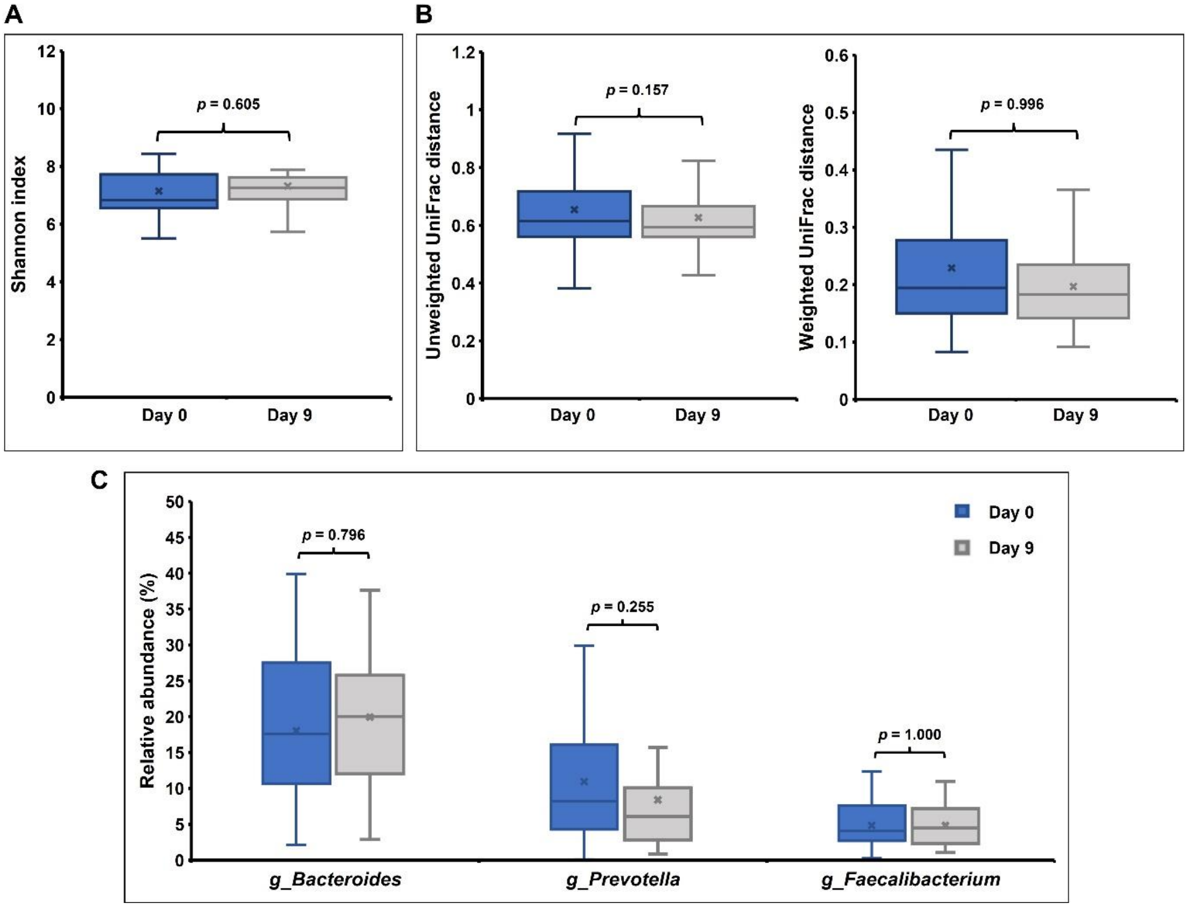

Figure S4. Gut microbiota composition between Day 0 (Pretreatment) and Day 9 (Post-MMF treatment but pre-TMP-SMX treatment). (A) Boxplot of gut microbial α-diversity (Shannon index); (B) Boxplot of gut microbial β-diversity (Unweighted and weighted UniFrac distance). The box represents the interquartile range (IQR) between the first and third quartiles, and the line and cross inside the box represent the median and mean, respectively. (C) Relative abundance of the three main abundant genera in the 16 study participants between Day 0 (pretreatment) and Day 9 (post-MMF treatment but pre-TMP-SMX treatment). *p*-value determined the significant difference after statistical corrections using the original false discovery rate (FDR) method of Benjamini-Hochberg for multiple comparisons.
