# Supplementary material for: Gut Microbiota-Mediated Pharmacokinetic Drug–Drug Interactions between Mycophenolic Acid and Trimethoprim-Sulfamethoxazole in Humans
Source: Pharmaceutics. 2023 Jun 14;15(6):1734. doi: 10.3390/pharmaceutics15061734 (PMC10304707; doi:10.3390/pharmaceutics15061734)
Supplement: Supplementary file 1 [file pharmaceutics-15-01734-s001.zip › Method S1.pdf]

Supplementary Method

Method S1. The whole-exome-based single nucleotide polymorphisms (SNPs) phylogenetic analyses

We collected blood samples from the study participants, prepared PBMC, and froze the samples at -80oC before the samples were extracted. The genomic DNA was extracted from PBMC using a modified salting-out extraction (1). Genomic DNA was quantified and qualified via nanodrop® (Thermofisher Scientific) and electrophoresis, 0.8% of agarose gel with a 1 kb ladder. After that, genomic DNA was sent to Illumina whole-exome sequencing in Macrogen (South Korea). The standard Illumina protocols and Illumina paired-end adapters were used for library preparation from the fragment genomic DNA. Sequencing libraries were constructed with 300 - 400 bp insert length and library preparation using SureSelect V7-post. WES was performed using the Illumina Novaseq platform with a standard 150 bp paired-end read. Mean target coverage of 100X for all samples.

The raw sequence of each sample was checked for quality using FASTQC version 0.11.9 (2). Then cut the adapter from the read using Cutadapt software (3). After that, we used the CLC genomic workbench 20.0 (QIAGEN) (www.qiagenbioinformatics.com) for variant calling. The workflow of this pipeline mapped the raw reads of each sample with the human reference genome (GRCh37 or hg19). The default parameter setting included the minimum read length of 20, minimum coverage of 10, minimum count of 2, minimum frequency (%) of 5, and pass base quality filter.

Phylogenetic analyses were performed on two sets of single nucleotide polymorphisms (SNPs) detected in a set of the 681 genes involved in human gut microbiota (4-8) of the study participants and a subset of specific 340 immune genes within the 681 genes. The two multi-sample VCF files of both gene sets were converted to FASTA format files of multiple

sequences alignment (MSA) using VCF-kit (9). For the phylogenetic construction, the best-fit nucleotide substitution model finding, and the maximum likelihood tree estimation were performed on both MSA files using IQ-TREE2 (10-12). IQ-TREE2 was programmed to write bootstrap trees with branch lengths and perform ultrafast bootstrap to generate 1,000 bootstrap trees. The resulted phylogenetic trees were, then, visualized using FigTree V1.4.4 (http://tree.bio.ed.ac.uk/software/figtree/).

References

1. Mohammadpour A. Evaluation of a modified salt-out method for DNA extraction from whole blood lymphocytes: A simple and economical method for gene polymorphism. Pharmaceutical and Biomedical Research. 2018;4(2):28-32.
2. Andrews S. FastQC: a quality control tool for high throughput sequence data. Babraham Bioinformatics, Babraham Institute, Cambridge, United Kingdom; 2010.
3. Martin M. Cutadapt removes adapter sequences from high-throughput sequencing reads. EMBnet journal. 2011;17(1):10-2.
4. Hall AB, Tolonen AC, Xavier RJ. Human genetic variation and the gut microbiome in disease. Nature Reviews Genetics. 2017;18(11):690-9.
5. Spor A, Koren O, Ley R. Unravelling the effects of the environment and host genotype on the gut microbiome. Nature Reviews Microbiology. 2011;9(4):279-90.
6. Goodrich JK, Davenport ER, Beaumont M, Jackson MA, Knight R, Ober C, et al. Genetic Determinants of the Gut Microbiome in UK Twins. Cell Host Microbe. 2016;19(5):731-43.
7. Blekhman R, Goodrich JK, Huang K, Sun Q, Bukowski R, Bell JT, et al. Host genetic variation impacts microbiome composition across human body sites. Genome Biology. 2015;16(1):191.

8. Lopera-Maya EA, Kurilshikov A, van der Graaf A, Hu S, Andreu-Sánchez S, Chen L, et al. Effect of host genetics on the gut microbiome in 7,738 participants of the Dutch Microbiome Project. Nature Genetics. 2022;54(2):143-51.

9. Cook DE, Andersen EC. VCF-kit: assorted utilities for the variant call format. Bioinformatics. 2017;33(10):1581-2.

10. Minh BQ, Schmidt HA, Chernomor O, Schrempf D, Woodhams MD, von Haeseler A, et al. IQ-TREE 2: New Models and Efficient Methods for Phylogenetic Inference in the Genomic Era. Mol Biol Evol. 2020;37(5):1530-4.

11. Kalyaanamoorthy S, Minh BQ, Wong TKF, von Haeseler A, Jermiin LS. ModelFinder: fast model selection for accurate phylogenetic estimates. Nat Methods. 2017;14(6):587-9.

12. Hoang DT, Chernomor O, von Haeseler A, Minh BQ, Vinh LS. UFBoot2: Improving the Ultrafast Bootstrap Approximation. Mol Biol Evol. 2018;35(2):518-22.
